# Genes at the Crossroad of Primary Immunodeficiencies and Cancer

**DOI:** 10.3389/fimmu.2018.02544

**Published:** 2018-11-01

**Authors:** Charlotte Derpoorter, Victoria Bordon, Geneviève Laureys, Filomeen Haerynck, Tim Lammens

**Affiliations:** ^1^Pediatric Hematology-Oncology and Stem Cell Transplantation, Ghent University Hospital, Ghent, Belgium; ^2^Center for Primary Immune Deficiency Ghent, Ghent University Hospital, Ghent, Belgium; ^3^PID Research Laboratory, Ghent University, Ghent, Belgium; ^4^Cancer Research Institute Ghent, Ghent, Belgium

**Keywords:** primary immunodeficiency, cancer, predisposition, genetics, biomarkers

## Abstract

Primary immunodeficiencies (PIDs) are a heterogeneous group of inherited disorders affecting one or multiple components of the innate and/or adaptive immune system. Currently, over 300 underlying genetic defects have been discovered. The most common clinical findings in patients with PIDs are infections, autoimmunity, and malignancies. Despite international efforts, the cancer risk associated with PIDs, given the heterogeneous character of this group of diseases, is difficult to estimate. The diverse underlying mechanisms of cancer in PID add another layer of complexity. Treatment of cancer within a context of PID is complicated by serious toxicities and long-term effects, including second malignancies. This review will focus on the little-known crossroad between PID and cancer genes and the value thereof for directing future research on our understanding of cancer in PID and for the identification of early cancer biomarkers in PID patients.

## Introduction

Integrity of the immune system is crucial in the defense toward infectious organisms and surveillance on deviating cellular transformations, i.e., development of cancer. Primary immunodeficiency diseases (PIDs) constitute a heterogeneous group of life-threatening heritable genetic disorders in which parts of the human immune system are missing or dysfunctional (1). Per definition, PIDs are thus characterized by an increased susceptibility to infections, autoimmunity, inflammatory organ damage, and malignancy (2–4). During the last two decades, driven by technological advances in next-generation sequencing, progress has been made in defining the genetics of PID (5). Nowadays, more than 300 PID-causing genes are reported (6), classified into eight categories based on the affected immune function.

An increased risk for malignancy in PIDs has been recognized for many years (7–11). Moreover, the presence of a “malignancy” has been acknowledged as a diagnostic criterion for some PIDs by the European Society of Immunodeficiencies (ESID) (https://esid.org) and malignancy is the second leading cause of death in PID patients. In general, an excess of cancer risk in PID patients compared with an age-adjusted population is observed for all cancer types. As “common variable immunodeficiency” (CVID) is the most common PID subtype, incidence results are often focused on this subgroup, revealing a higher incidence for lymphoma and an association with stomach and skin cancer. This increased risk is likely multifactorial and related to viral infections and/or sustained activation and proliferation during chronic infections causing genetic instability in lymphocytes (12, 13). The enhanced risk for gastric cancer has been attributed to *Helicobacter pylori* infection, although the exact mechanisms are still unknown (14). Cancer incidences for other PID subtypes are not well-defined, but associations were noted for lymphoma, gastric cancer, skin cancer and/or leukemia in Ataxia Telangiectasia (AT), “diseases of immune dysregulation” and “other well-defined immunodeficiency syndromes” (8, 9, 14–16).

The most commonly accepted theory to explain an enhanced cancer risk in PID patients is based on the reduced cancer surveillance caused by PID mutations (17, 18). This view has recently been challenged (12) and it must be considered that PID genetic defects *per se* alter the risk for malignant transformation through a direct oncogenic effect, exemplified by DNA repair disorders. In addition, PID genes cause altered T- and B-cell functions through impaired V(D)J recombination, class switch recombination and somatic hypermutation, causing chronic viral infections and inflammation (19). Similarly, researchers have shown that Natural Killer T (NKT) cells might play a major role in tumor development in a genetic background susceptible to carcinogenesis (20), as it has been observed that loss of type 1 NKT cells enhances tumor development in p53^+/−^ mice and secondly, NKT cells protect against B-cell lymphoma development in mice (20, 21). A comprehensive overview of the mechanisms that may explain the enhanced risk of cancer is out of scope of this review, and has recently been documented by Hauck et al. (13).

Within this review, we provide a synopsis on the current knowledge about the genetics of malignancies in PID. In addition, we will elaborate on the presence of a largely ill-explored intersection between PID and cancer genes and the importance thereof for guiding future research on our understanding of cancer in PID and for the identification of early cancer biomarkers in PID patients.

## Intersection of PID and cancer genetics

The study by Neven et al. is unique in extensively documenting molecular and immunophenotypical resemblance between lymphomas in patients with *IL10* and *IL10R* loss-of-function mutations (causing severe early-onset inflammatory bowel disease) and germinal center B-cell diffuse large B-cell lymphoma (GCB DLBCL) (22). Although typical DLBCL mutations were observed (including the mutation p.S219C in *MYD88*), mutations in histone and chromatin modifying genes were completely absent, in contrast to classical DLBCL (22). Additional gene expression profiling revealed some similarities, but also enriched expression of spliceosome pathway genes and genes involved in ubiquitin-mediated proteolysis was present in PID-associated, but not sporadic, DLBCL.

**Figure 1:**
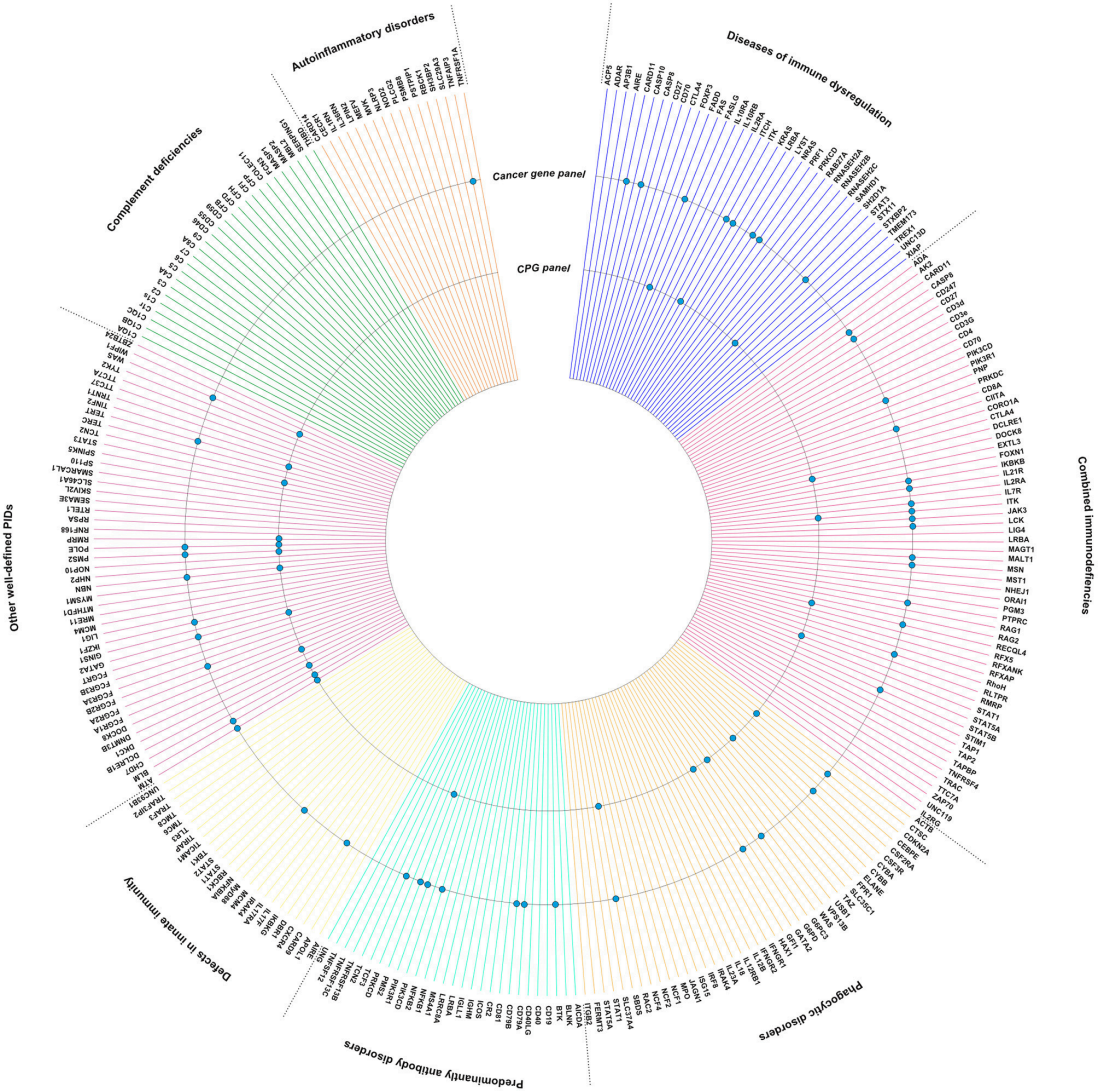
Intersection between PID genes (https://esid.org) and the Cancer Gene Census (CGC), a catalog of genes which contain mutations that have been causally implicated in cancer (https://cancer.sanger.ac.uk/census) (24), or Cancer Predisposition Genes (CPGs) (23). PID genes also listed in the CGC or as CPG are indicated with a dot.

Although broad biological insights into the pathogenesis and characteristics of PID-associated cancers remain scarce, it is notable that many key molecules going awry in PID, have been mentioned independently in the context of carcinogenesis. In order to strengthen these observations, we have visualized the intersection of PID-causing genes (https://esid.org) with true cancer genes (https://cancer.sanger.ac.uk/census) and cancer predisposition genes (23) (Figure 1). It is important to note that different mutations in the same gene can lead to varying clinical phenotypes. There is a need to characterize the mutational landscape in sporadic cancer compared to PID-associated cancer and additionally in PID patients with a high cancer risk compared to those with a low risk.

## PID and cancer predisposition genes

Interestingly, several well-known PID genes are also recognized as cancer predisposition genes, such as *GATA2* and *BLM*. ***GATA2*** is a key transcription factor required for the development and maintenance of hematopoietic stem cells. The phenotype of *GATA2* mutations comprises MonoMAC syndrome (PID associated with disseminated non-tuberculous mycobacterial infections) and familial myelodysplastic syndrome (MDS) (25–27). However, one should note that mutations are documented in different domains according to the clinical phenotypes: MDS/Acute Myeloid Leukemia (AML)-associated mutations are located in the zinc finger motif ZF2, whereas PID-associated mutations mostly before ZF2. Positive testing of germline *GATA2* mutations in leukemia has profound effects on clinical management, such as adapted prophylactic antimicrobial management during therapy (27). Importantly, screening of familial donors for *GATA2* mutations is crucial in the procedure for hematopoietic stem cell transplantation, the only available therapy. Similarly, the ***BLM* gene**, coding for a DNA helicase involved in DNA repair, has a well-described role in both cancer predisposition and immunodeficiency (28). DNA repair is crucial in the generation of B- and T-cell antigen receptors through T-and B-cell-specific V(D)J rearrangements, class switch recombination and/or somatic hypermutation. Defects in *BLM* thus impair lymphocyte development, explaining the immunodeficiency phenotype. In addition, through its role in maintaining genomic stability, an increased cancer risk is observed in these patients (12, 13, 28). *FAS, ITK, RECQL4, CDKN2A, WAS, SBDS, ATM, NBN*, and *POLE* are other examples of PID-causing genes involved in genetic cancer predisposition (29–32).

## PID and cancer genes

Next to these well-known relations, Figure 1 also illustrates that several cancer genes, not yet officially recognized within predisposition panels, are also germline mutated in PIDs. As PID is a hallmark of cancer predisposition, one might speculate that several of the genes listed within the cancer gene list and intersecting with the PID list are potentially undiscovered or underexplored cancer predisposition genes (Figure 1). This is obviously the case for genes such as *IKZF1, TYK2, MYD88*. It indeed has been proven that several of the genes known to be somatically mutated in cancer types (i.e., *IKZF1* in leukemia), are found to be germline mutated through i.e., familial cancer studies, and thus getting recognized as cancer predisposition genes (33–35). This indicates that immunologists should acknowledge the possibility of an underlying cancer predisposition in PID with those genes affected, while vice versa oncologists should be triggered to evaluate a potential underlying PID upon a novel cancer diagnosis.

Somatic defects in ***IKZF1***, a hematopoietic zinc finger transcription factor, have been linked to acute lymphoblastic leukemia (ALL) for several years and have been proven to harbor negative prognostic effects (36). Recently, *IKZF1* mutations have been identified in familial ALL and in the germline of presumably sporadic cases. These mutations were dispersedly distributed over the whole protein coding sequence and were proven to be functionally damaging, even when not located in one of the functional domains. In the index family, individuals without ALL, but carrying the D186fs mutation in *IKZF1*, had variable lymphopenia and low-normal IgG levels, albeit not defined as immunodeficient (33). Remarkably, germline *IKZF1* mutations within the ZF2 DNA binding domain were reported to be associated with an early-onset CVID (37). Similarly, an intersection between PID and cancer genetics can be observed for mutations in several of the **JAK-STAT** signal pathway genes (*STAT3, STAT5B*). Indeed, somatic mutations in *STAT* and *JAK* family members have been recognized as important drivers in oncogenesis, especially in different leukemia types (38–40). In addition, germline mutations in JAK-STAT signaling are associated with PIDs. Notably, the PID phenotypes depend on the affected gene and mutation, ranging from mild phenotypes involving *TYK2*, a moderate hyper-IgE syndrome for *STAT3* and severe combined immunodeficiency (SCID) in case of *JAK3* mutations (41). It is only recently, that mutations in *TYK2*, a JAK kinase family member, were found in the germline of patients presenting with a second primary leukemia, causing constitutive JAK signaling and a propensity for developing leukemia (42).

Also, for ***MYD88*** a role in immunodeficiency and cancer development has been shown. MYD88 deficient animals have an increased risk of gastric cancer upon challenging them with *H. pylori* (43). In humans, deficiency in MYD88 results in impaired TLR signaling (44, 45). These patients have recurrent invasive infections (cellulitis, sepsis, meningitis, osteomyelitis), mainly caused by *Staphylococcus aureus* and *Streptococcus pneumoniae*. In addition, *MYD88* is found to be mutated in several hematologic B-cell malignancies, as Waldenström macroglobulinemia, DLBCL and IgM monoclonal gammopathy (46).

## Other PID genes

Importantly, it has to be recognized that several important gaps need to be filled. This is illustrated by the involvement of the ***CTLA4*** in both cancer and PIDs (47–49). Indeed, a survey of 131 affected *CTLA4* mutation carriers shows a cancer prevalence of 12.9%, mainly lymphoma, gastric adenocarcinoma and metastatic melanoma (50). Nevertheless, *CTLA4* has not been mentioned in the cancer census gene list.

**Figure 2:**
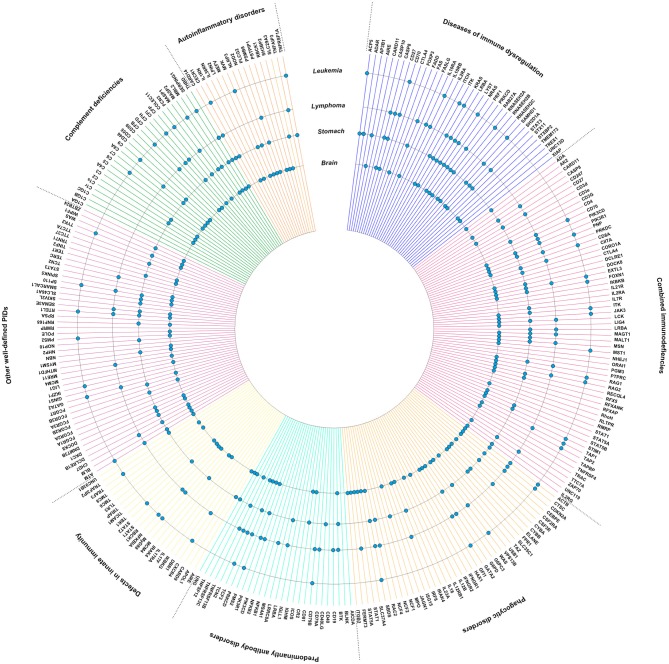
PID genes (https://esid.org) associated with high impact mutations in lymphoma, leukemia, brain tumors, and stomach cancer. Data was generated using the International Cancer Genome Consortium (ICGC) Data Portal, a catalog of genomic abnormalities from over 20,000 tumor genomes (https://dcc.icgc.org/) (51). Donor age at diagnosis was restricted to age categories with an increased risk for PID-associated malignancies (0–59 years) (8, 11). PID genes with high impact mutations found in lymphoma, leukemia, brain tumors, and/or gastric cancer are indicated with a dot.

Subsequently, we visualized the intersection between PID genes and genes published to be somatically mutated in lymphoma, leukemia, stomach cancer and brain tumors (Figure 2). Notably, mutations found in lymphoma are clearly enriched in PID genes involving “primary antibody disorders” (PADs) and “diseases of immune dysregulation,” and less frequent in “defects in innate immunity.” Although differences are smaller, fewer somatically mutated genes can be observed in PADs and “phagocytic disorders” for lymphoma, and in PADs and “defects in innate immunity” for brain tumors. Of note is the high intersection between PID genes and genes mutated in brain tumors (Figure 2). The high level of intersection might partially result from the observation that hypermutation is especially found in brain tumors, specifically the H3.3 or H3.1 K27-wildtype high-grade gliomas with biallelic germline mutations in *MSH6* or *PMS2* (52).

## Discussion

The diagnosis and management of cancer in PID patients is cumbersome. Guidelines and techniques for cancer screening within PID are ill-defined and should be evaluated in large international study cohorts. Although our understanding of the mechanisms of cancer development in PID is increasing, the genetic and molecular characteristics of cancers in PID patients remain uncovered. Here, we show that several PID genes are recognized as cancer predisposition genes. In addition, several of the genes listed within the cancer gene list and intersecting with the PID list are potentially undiscovered or underexplored cancer predisposition genes. Although the specific mutations and thus functional impact in both entities might be different, this observation implies that both oncologists and immunologists should be triggered to search for an underlying PID or potential development of cancer, respectively. Importantly, many PID genes might be candidates for further study in cancer research.

Improved understanding in cancer biology has led to the development of immunotherapies. The contribution of germline genetic factors is expected to be higher in pediatric cancers (53, 54) and PIDs. One could question if current immunotherapies might improve clinical outcomes for pediatric cancer. However, studies have illustrated that current inhibitory checkpoint immunotherapies are most efficient for tumors with high mutational load, which is not the case for most pediatric malignancies (55, 56). In this respect, there is a need for novel targets, again highlighting the importance of elucidating the genetics of PID-associated cancers in children, which may contribute to novel targeted treatment.

Importantly, efforts in creating awareness will be crucial to obtain these goals. Together with increasing technological advances (including i.e., testing cancer patients on radiosensitivity), one could expect to see the number of PID patients growing, especially if cancer is the first manifestation. This increasing number unavoidably will impact our view on the cancer landscape and incidence within PID. In addition, PID patient registers should be established/maintained with sufficient information on underlying genetic defects and malignancies or, ideally, an intersection with a national cancer registry. Furthermore, it is of utmost importance to improve the collection of biological material of PID patients with associated malignancy and perform “omic” studies to enhance our knowledge on this specific disease biology, improve on diagnosis and follow-up, and design newer therapeutic options.

## Conclusion

Despite international efforts, the cancer risk associated with PIDs is difficult to estimate. Furthermore, treatment of cancer within a context of PID is complicated by serious toxicities and long-term effects, including second malignancies. Detailed molecular studies are required to identify common and distinct molecular pathways in PID-associated malignancies vs. sporadic cases and in PID patients with a high cancer risk vs. those with a low risk. These biological insights may allow early molecular recognition of cancer in PID, optimization of existing therapies and the development of targeted therapies, reducing toxicities within this patient population.

## Data availability statement

The datasets analyzed for this study can be found in the ICGC Data Portal (https://dcc.icgc.org/).

## Author contributions

TL, CD, and FH conceptualized the study. TL and CD generated the figures. All authors contributed to the writing of the manuscript and approved the final version of the manuscript.

### Conflict of interest statement

The authors declare that the research was conducted in the absence of any commercial or financial relationships that could be construed as a potential conflict of interest.
